# Does early identification of high work related stress affect pharmacological treatment of primary care patients? - analysis of Swedish pharmacy dispensing data in a randomised control study

**DOI:** 10.1186/s12875-020-01140-x

**Published:** 2020-04-25

**Authors:** Pernilla J. Bjerkeli, Ingmarie Skoglund, Kristina Holmgren

**Affiliations:** 1 School of Health and Education, University of Skövde, PO Box 408, SE-521 28 Skövde, Sweden; 2 Department of Primary Health Care, Institute of Medicine, Sahlgrenska Academy, University of Gothenburg, PO Box 454, SE-405 30 Gothenburg, Sweden Sweden; 3 The Research and Development Department, Region Västra Götaland, Södra Älvsborg, Borås, Sweden; 4 Department of Health and Rehabilitation, Institute of Neuroscience and Physiology, Sahlgrenska Academy, University of Gothenburg, PO Box 455, SE-405 30 Gothenburg, Sweden

**Keywords:** Work related stress, Stress, Medication use, Pharmacy dispensing data, Intervention

## Abstract

**Background:**

The study is part of a randomised controlled trial with the overall aim to evaluate if use of the Work Stress Questionnaire (WSQ), combined with feedback at consultation, can be used by healthcare professionals in primary health care to prevent sickness absence. The specific aim of the present study was to investigate whether there were differences in pharmacy dispensing of prescription medications between the intervention group and the control group.

**Methods:**

The study was a randomized controlled trial. Non-sick-listed employed women and men, aged 18 to 64 years, seeking care at primary health care centres (PHCCs) were eligible participants. The intervention included early identification of work-related stress by the WSQ, general practitioner (GP) training and GP feedback at consultation. Pharmacy dispensing data from the Swedish Prescription Drug Register for a period of 12 months following the intervention was used. Primary outcomes were the number of different medications used, type of medication and number of prescribing clinics. Data was analysed using Mann Whitney U tests and chi-square tests.

**Results:**

The study population included 271 individuals (132 in the intervention group and 139 in the control group). The number of different medications used per individual did not differ significantly between the control group (median 4.0) and the intervention group (median 4.0, *p*-value 0.076). The proportion of individuals who collected more than 10 different medications was higher in the control group than in the intervention group (15.8% versus 4.5%, *p* = 0.002). In addition, the proportion of individuals filling prescriptions issued from more than three different clinics was higher in the control group than in the intervention group (17.3% versus 6.8%, *p* = 0.007).

**Conclusion:**

Systematic use of the WSQ combined with training of GPs and feedback at consultation may affect certain aspects of pharmacological treatment in primary health care patients. In this randomised control trial, analysis of pharmacy dispensing data show that patients in the intervention group had less polypharmacy and filled prescriptions issued from a smaller number of different clinics.

**Trial registration:**

ClinicalTrials.gov. Identifier: NCT02480855. Registered 20 May 2015.

## Background

According to the WHO, work-related stress is defined as the response people may have when presented with work demands and pressures that are not matched to their knowledge and abilities and which challenge their ability to cope [1]. The organizational climate is an important factor [2]. A large European survey, including Sweden, published in 2009 indicated that the prevalence of work related stress among working Europeans was 22% [3]. Other studies have shown similar results, depending on the definitions and measurements used [4]. The European survey [3], showed that the prevalence of work related stress was higher than average in Sweden (38%). Sweden has a relatively high labour market participation, including high proportions of women and elderly workers [5]. In a Swedish study, a large proportion of working women reported high perceived stress owing to different factors related to their work [6]. Ten percent reported stress due to indistinct organization, 25% due to individual demands and commitment, 22% due to low influence at work and 33% due to work interference with leisure time [6].

Stress is a known risk factor for poor health [7, 8],. Several diseases and symptoms have a known association with stress, for example depression [8], other mental health problems [9], shoulder and neck pain [10] other musculoskeletal disorders [11], and cardiovascular disease [8, 12]. Studies also suggest associations between stress and upper respiratory tract infections, asthma, herpes viral infections, autoimmune diseases, wound healing and impaired neuropsychological function [8, 13, 14]. Long-term perception of stress can also constitute a risk for sickness absence [6, 15], which in turn does not only mean poor economy and suffering for the individual and costs for society; employers also risk drops in production, loss of competence and costs for new employees [16].

A global, systematic review examining the cost of work-related stress recently concluded that, apart from loss in productivity, costs for use of health care and medications were the largest cost related to work related stress [17]. Use of health care and medications constituted around 10–30% of the total cost. Despite this, few studies have investigated pharmacological treatment among those suffering from work related stress. A cross-sectional study among French community pharmacy employees indicated that work related stress was associated with medication use [18]. In line with this, an Italian study found that work related stress was associated with increased use of antidepressant medication [19]. Apart from this, little is known about medication use in relation to work related stress.

The present study is part of a randomised controlled trial with the overall aim to evaluate if a systematic use of the Work Stress Questionnaire (WSQ) combined with feedback at consultation, could be used by healthcare professionals in primary health care to prevent sickness absence [20, 21]. Prior publications from the study are the study protocol [21] and an analysis the interventions effect on sickness absence [21]. The present study was focused towards analysis of how the intervention affected pharmacological treatment of patients. The aim was thus to investigate whether there were differences in pharmacy dispensing of prescription medications between the intervention group and the control group.

## Methods

The study is a randomised controlled trial [20], which is part of the TIDAS-project within the New Ways research program at the Sahlgrenska Academy, University of Gothenburg, Sweden. The project has been approved by the Regional Ethical Review Board in Gothenburg (Ref. No. 125–15). The randomised control trial was designed in accordance with CONSORT recommendations [22]. Trial registration: ClinicalTrials.gov. Identifier: NCT02480855. Registered 20 May 2015. The details of the data collection have been more closely described in the study protocol [20].

### Procedure

Data was collected at seven different primary health care centres (PHCCs) (five public and two private) in the region Vastra Gotaland, Sweden. The general practitioners (GPs) at the participating PHCCs who consented to take part in the study were randomised into either intervention (systematically using the WSQ and providing feed back to the patients during consultation) or control (treatment as usual).

The randomization was done by writing the names of all GPs at the participating PHCC on individual slips of paper. The papers were folded and placed in a nontransparent bowl. Colleagues that were not involved in the study drew the names one at a time, and the names were alternately included in the intervention or the control group. The GPs who were randomised into the intervention group received training in how to use and interpret the WSQ and how to give feedback to participants. They also got information about the relationship between work related stress and health and about referrals. The brief intervention has been developed in close dialogue with colleagues in primary health care. The data collection took place between May 2015 and January 2016. At each PHCC, the data collection lasted 4–8 weeks (except for one PHCC, where it took 12 weeks). The time of 4–8 weeks was selected based on experience from earlier research projects in primary health care. A research assistant was placed at the PHCC, and together with personnel at the PHCC, recruited consecutively eligible patients. All potential participants were given information about the study, including information about record linkage to registers. Written informed consent was obtained from all participants.

All participants were asked to fill out a questionnaire containing background questions and the WSQ at baseline. The WSQ consists of 21 main items, which are grouped into four categories [23]. The categories are perceived stress due to *indistinct organization and conflicts,individual demands and commitment, influence at work* and, *work interference with leisure time.* The reliability and face validity of the WSQ has been tested [23].

In the intervention group, the WSQ-score was given to the GP before he or she met the patient. During the consultation, the GP provided feed back to the patient based on the results of the WSQ and, if relevant, measures to prevent sick leave were discussed. In the control group, the patient answered the questionnaire after the consultation and the GPs were thus not aware of whether the patient took part of the study or not.

### Participants

Eligible participants were employed men and women aged 18–64 years who visited a GP at one of the appointed PHCCs during the study period due to mental and/or physical health complaints, including depression, anxiety, musculoskeletal disorders, gastrointestinal, cardiovascular symptoms and other stress-related symptoms. Patients who were already sick listed and women who were pregnant were not included. Nor were patients seeking care for diabetes, urinary tract infections, infections, chronic obstructive lung disease, fractures, lump and spots, allergy and psychiatric diagnoses such as schizophrenia, other psychoses or bipolar diagnoses, as well as medical check-ups.

Because the sample size was calculated based on the primary endpoint of the RCT, the sample size for this secondary analysis was not calculated. [20]. The calculation was performed to determine the number of participants needed to detect at least a 15% difference between the intervention group and the control group concerning the number of registered sick leave days during 12 months after inclusion. With a two-sided t test, statistical significance of *p* < 0.05 and 80% power, at least 135 participants were needed in each group. The variable registered sick leave was chosen since it was the primary outcome of the randomized control trial [20].

### Data

The questionnaire that patients filled out at the PHCCs provided data concerning age, sex, other background variables and the WSQ.

Individual level data concerning medication use was collected from the Swedish Prescribed Drug Register (SPDR) [24], using the unique personal identification number that all Swedish residents hold. The SPDR contains information on all pharmacy dispensing’s of prescription medications made in Sweden. For each participant, information on prescription fills during a period of 365 days after the intervention date was included. We obtained information on the dispensed medication (product, Anatomical Therapeutic Chemical Classification (ATC)-code, amount and date of purchase) and the prescriber (work place).

The National Patient Register (NPR) was used to obtain information on hospital care episodes (hospitalisation) during the study period. This was necessary since the SPDR does not contain information about medication use in hospitals and a hospital stay might otherwise be misclassified as a period without medication use. Information about deaths among the participants during the study period (death date) was retrieved from the Causes of Death register. All participants were alive throughout the study period. All registers used in this study are managed by the Swedish Board of Health and Welfare.

### Assessment of variables

A participant was categorized as a user of a specific medication if he or she had made at least one prescription fill of the medication during the study period. The number of different medications used per individual during the study period was summarised and categorized into 0, 1–4, 5–10 and > 10 different medications. Medications with different ATC-codes on the seven digit level were classified as different. The cut-off at 5 different medications is one of the most commonly used numerical definitions for polypharmacy [25] and the cut of at ten different medications was selected to identify major polypharmacy. Used medications were also presented in groups defined by ATC-codes on a four digit level.

Information about prescribing clinic for each prescription was used to gather information about the number of different prescribing clinics involved for each patient during the study period. This variable was categorized into 0, 1, 2–3 and > 3 different clinics.

### Statistical analysis

The distribution of sex and age between the intervention and control group was analysed by calculating the 95% confidence interval (95% CI) for the difference in proportions, using the following formula.

$$ 95\% CI={p}_1-{p}_2\pm 1.96\ast \surd \Big(\left({p}_1\ast \left(1-{p}_1\left)\right)/{n}_1\right)+\left(\left({p}_2\ast \left(1-{p}_2\right)\right)/{n}_2\right)\right) $$

where n is the total number of observations and p is the proportion of observations with the characteristic of interest. Confidence intervals not including zero indicated a significant difference in proportions. The significance level was set to 0.05. The same formula and analysis were applied to compare the use of different types of medication in the control group and in the intervention group. Results are presented for the ten ATC-groups that had the largest number of users in the study population.

The continuous outcome variables, number of different medications used and number of prescribing clinics, were tested for normal distribution using using the Shapiro-Wilk test. As they were both skewed, the Mann Whitney U test was used to compare medians. For the categorised versions of these variables, chi-square tests were used to test each category’s distribution between the intervention- and control group. The significance tests for these variables (Mann Whitney U tests and chi-square tests) were interpreted using Bonferroni adjusted alpha levels of 0.01 per test (0.05/5), since five tests were performed per variable (four categories and the continuous variable).

All data management and statistical analysis was performed with IBM SPSS Statistics 24.

## Results

The study population included 271 primary health care patients (132 in the intervention group and 139 in the control group), Table 1. The control group had a lower proportion of individuals in the 50–65 years age group, Table 1. Apart from that, age and sex were equally distributed across the two groups. More detailed information about distribution of background variables between the intervention and control group has been published in the study protocol [20].

**Table 1:** *Study population in a randomised controlled trial at Swedish primary heath care centres. The confidence intervals indicate the 95% confidence interval for the difference between the proportion in the intervention group (n = 132) and in the control group (n = 139)*

	Total	Intervention		Control		Difference in proportion	95% CI for difference in proportion
		n	%	n	%		
**Total number of patients**	271	132	100.1-	139	100.0-		
**Sex**							
Women	185	88	66.7	97	69.8	3.1	−0.080-0.14
Men	86	44	33.3	42	30.2	3.1	−0.080-0.14
**Age (years)**							
18–29	45	20	15.2	25	18.0	2.8	−0.061-0.12
30–49	127	55	41.7	72	51.8	10.1	−0.22-0.017
50–65	99	57	43.2	42	30.2	13	0.016–0.24
**Number of general practitioners**	63	29	–	34	–		

A Mann Whitney U test indicated that the number of different medications used per individual did not differ significantly between the control group (median 4.0) and the intervention group (median 4.0), U = 8036,0, p-value 0.076). A total of 26 participants did not fill any prescription at all during the study period (365 days), 17 in the intervention group and 9 in the control group, Table 2. The proportion of individuals who collected more than 10 different medications was higher in the control group (15.8% versus 4.5%, *p* = 0.002), Table 2.

**Table 2:** *Number of different medications used and number of different prescribing clinics per individual during one year in a sample of 271 primary health care patients. One prescription fill during the study period (365 days) was enough to be classified as a user of a medication. Values are results of chi-square tests performed separately for each category. P*-values should be interpreted using the Bonferroni adjusted alpha level of 0.01

	Total	Intervention group		Control group			
	N	n	%	n	%	Χ^2^ (df.)	p
Total	271	132	100.0	139	100.1		
No. of different medications							
0	26	17	12.9	9	6.5	3.2 (1)	0.074
1–4	133	62	47.0	71	51.1	0.5 (1)	0.50
5–10	84	47	35.6	37	26.6	2.6 (1)	0.11
> 10	28	6	4.5	22	15.8	9.3 (1)	0.002
No. of different prescribing clinics							
0	26	17	12.9	9	6.5	3.2 (1)	0.074
1	102	46	34.8	56	40.3	0.8 (1)	0.36
2–3	110	60	45.5	50	36.0	2.5 (1)	0.11
> 3	33	9	6.8	24	17.3	6.9 (1)	0.007

During the study period, 33 participants filled prescriptions that were prescribed from more than three different clinics, Table 2. This proportion was significantly higher in the control group than in the intervention group (17.3% versus 6.8%, *p* = 0.007). The median number of different prescribing clinics per individual did not differ significantly between the control group (median 2.0) than in the intervention group (median 2.0, U = 8403.5, *p* = 0.21).

In Table 3, the proportion of individuals having filled at least one prescription of a medication from the top ten most prevalent medication groups are shown for the intervention group and the control group, respectively. The most prevalent medication groups in the study population were anti-inflammatory and antirheumatic products (*n* = 92), antidepressants (*n* = 91) and drugs for treatment of peptic ulcer (*n* = 58).

**Table 3:** The 10 most prevalent prescription medication groups purchased by 271 primary health care centre patients in a randomised controlled trial. One prescription fill during the study period (365 days) was enough to be classified as a user

Name of medication group		Total No. users	Users in the intervention group (***n*** = 132)		Users in the control group (***n*** = 139)		Difference in proportion	95% CI for difference in proportions	
		N	n	%	n	%		upper	lower
M01A	Antiinflammatory and antirheumatic products, non-steroids	92	41	31.1	51	36.7	5.6	−0.05	0.17
N06A	Antidepressants	91	41	31.1	50	36.0	4.9	−0.06	0.16
A02B	Drugs for treatment of peptic ulcer	58	25	18.9	33	23.7	4.8	−0.05	0.15
N02B	Other analgesics and antipyretics	57	27	20.5	30	21.6	1.1	−0.08	0.12
N05B	Anxiolytics	53	25	18.9	28	20.1	1.2	−0.08	0.10
J01C	Beta-lactam antibacterials, penicillins	49	27	20.5	22	15.8	−4.7	−0.13	0.05
N05C	Hypnotics and sedatives	49	21	15.9	28	20.1	4.2	−0.05	0.13
N02A	Opioids	44	22	16.7	22	15.8	−0.9	−0.10	0.08
C07A	Beta blocking agents	33	17	12.9	16	11.5	−1.4	−0.09	0.07
R06A	Antihistamines for systemic use	29	11	8.3	18	12.9	4.6	−0.02	0.12

## Discussion

In this randomised control trial, the intervention group had a smaller proportion of individuals filling prescriptions for as many as ten different medications or more (polypharmacy [25]) than the control group. In addition, the proportion of individuals filling prescriptions issued from more than three different clinics was lower in the intervention group than in the control group. These findings could be interpreted as a sign that the intervention has somehow affected the medication behaviour of the prescriber and/or the patient.

There are several potential mechanisms behind the possible effect of the intervention. The GPs in the intervention group were given training including knowledge about the relationship between psychosocial factors at work, stress, health, and sickness absence [20]. A systematic review concerning management of depression in primary health care has, however, indicated that strategies only including education are generally not effective [26]. In the present study, the GPs also received instructions on how to refer patients at risk to other health care providers such as physiotherapy, psychotherapy, occupational therapy and specialist care. Possibly, this training provided the GPs with other tools than prescription medication when managing patients. The increased awareness of these alternatives among the intervention GPs may have altered their prescribing behaviour. An alternative explanation is that the patients in the control group suffered from more disorders at base line and that they therefor needed a larger number of different medications. However, as previously published in the study protocol [20], no such differences could be observed. The only difference was a larger proportion of participants with musculoskeletal disorders in the intervention group then in the control group, thus rather suggesting a higher need for medication in the intervention group.

It is also possible that the use of the WSQ and the feedback at consultation have increased the patients’ awareness of their work related stress, thus inducing them to take measures to prevent stress from causing harm. This behaviour may be preventive of stress related disorders and thus reduce the patients need for medications. Previous research has shown that the WSQ can identify work related stress [23] and predict future sick leave [15] but direct effect on patient behaviour has not been studied.

The patients’ experience of the PHCC visit could also have been effected by the intervention thus altering the patients’ help seeking behaviour. Research has found that patients find the use of questionnaires helpful in communication with the GP, [27, 28] which might have contributed to increased confidence in relation to the GP and PHCC [29]. Patients in the intervention group were given feedback on their WSQ-score and they met with a GP who were most likely more aware of the relationship between psychosocial factors and health than usual and had more knowledge about potential referrals. The patients in the intervention group had filled prescriptions from a smaller number of different clinics than the control group patients. This could be interpreted as an indicator that they were more satisfied with their treatments and thus has less reason to visit other clinics.

### Methodological considerations

This study is based on pharmacy dispensing data [24]. This means that we do not know about all the prescriptions that were issued to the patient. We only know about the prescriptions that were actually filled in a pharmacy. It should also be noted that when using pharmacy dispensing data there is no information about whether the patients used the medication or not once they had collected it from the pharmacy.

A strength of this randomised control trial was that the intervention group and the control group were very similar in terms of age and gender [20]. The intervention group did, however, have a slightly larger proportion of participants in the highest age group (50 to 65 years) compared to the control group. This is important as medication use increases with increasing age [30]. As we found a higher proportion using more than ten different medications in the control group, where the proportion of older individuals was lower, it is possible that this has attenuated the effect of the intervention.

The main outcomes of this study were number of different medications, type of medication and number of different prescribing clinics. The number of different medications was used as an overall measurement of the patients medications use. The outcome was also used to identify polypharmacy [25]. A high score may indicate appropriate medication use in a patient with multiple disorders as well as inappropriate prescribing to patients who would actually benefit from using fewer medications. In this study we have not evaluated the appropriateness of the medication use. The second outcome, type of medication, was introduced in order to identify whether the intervention had different effect on the use of different types of medications. The top ten different medications used were similar to what can be seen in the general Swedish population [31] and did not differ significantly between the two groups. Possibly this is due to the limited sample size. Medications for psychiatric disorders and pain dominated in the study population. The third outcome was the number of different clinics from which the patient had filled prescriptions. It should be noted that this variable cannot identify all clinics that the patient had visited, it only includes clinics from which prescriptions were issued and later filled by the patient. A high score on this variable could either indicate that the patient has multiple disorders that cause him or her to visit several different specialists or it could indicate that the patient was not satisfied with the PHCC and thus visited another one. The intervention was not masked. It is, however, not likely that the participating GPs altered their prescribing behaviour simply as a result of knowing that they were taking part in the RCT since prescription medication use was not emphasised as a primary outcome of the study.

## Conclusion

Systematic use of the WSQ combined with training of GPs and feedback at consultation may affect certain aspects of pharmacological treatment in primary health care patients. In this randomised control trial, pharmacy dispensing data showed that patients in the intervention group had less polypharmacy and filled prescriptions issued from a smaller number of different clinics. Possible mechanisms could be that the training of the GPs provided them with other tools than pharmacological treatment and that the use of the WSQ increased awareness of psychosocial factors.

## Data Availability

The study uses data that were collected for this study specifically as well as data that were retrieved from a national pharmacy dispensing register. The datasets generated and analyzed during the current study are not publicly available due to restrictions in the ethical approval but are available from the corresponding author on reasonable request. The register data that support the findings of this study are available from the Swedish Board of Health and Welfare (www.socialstyrelsen.se/en/statistics-and-data/registers/) but restrictions apply to the availability of these data, which were used under license for the current study, and so are not publicly available. Data are however available from the authors upon reasonable request and with permission of the Swedish Board of Health and Welfare.

## Abbreviations

95% CI: 95% confidence interval
ATC: Anatomical therapeutic chemical classification
GP: General practitioner
PHCC: Primary health care centre
NPR: the national patient register
SD: Standard deviation
SPDR: the Swedish prescribed drug register
WSQ: the work stress questionnaire
